# The Role of β-Dystroglycan in Nuclear Dynamics

**DOI:** 10.3390/cells13050431

**Published:** 2024-02-29

**Authors:** Matthew Cook, Ben Stevenson, Laura A. Jacobs, Daniel Leocadio Victoria, Bulmaro Cisneros, Jamie K. Hobbs, Colin L. Stewart, Steve J. Winder

**Affiliations:** 1 School of Biosciences, University of Sheffield, Sheffield S10 2TN, UK; 2 A*STAR Skin Research Laboratories, Singapore 138648, Singapore; 3 Department of Genetics and Molecular Biology, Centro de Investigación y de Estudios Avanzados, Mexico City 07360, Mexico; bcisnero@cinvestav.mx; 4 Department of Physics and Astronomy, University of Sheffield, Sheffield S3 7RH, UK

**Keywords:** dystroglycan, muscular dystrophies, nucleus, AFM, myoblast

## Abstract

Dystroglycan is a ubiquitously expressed heterodimeric cell-surface laminin receptor with roles in cell adhesion, signalling, and membrane stabilisation. More recently, the transmembrane β-subunit of dystroglycan has been shown to localise to both the nuclear envelope and the nucleoplasm. This has led to the hypothesis that dystroglycan may have a structural role at the nuclear envelope analogous to its role at the plasma membrane. The biochemical fraction of myoblast cells clearly supports the presence of dystroglycan in the nucleus. Deletion of the dystroglycan protein by disruption of the *DAG1* locus using CRISPR/Cas9 leads to changes in nuclear size but not overall morphology; moreover, the Young’s modulus of dystroglycan-deleted nuclei, as determined by atomic force microscopy, is unaltered. Dystroglycan-disrupted myoblasts are also no more susceptible to nuclear stresses including chemical and mechanical, than normal myoblasts. Re-expression of dystroglycan in *DAG1*-disrupted myoblasts restores nuclear size without affecting other nuclear parameters.

## 1. Introduction

In skeletal muscle, dystroglycan and the associated dystrophin glycoprotein complex (DGC) functions to provide structural stability to the sarcolemma and as a signalling platform responsible for diverse functions including vasoregulation and focal adhesion assembly [1]. The dystroglycan pre-peptide is heavily modified by glycosyltransferases which confer the ability to bind the extracellular matrix (ECM). During dystroglycan (DG) processing, an integral Sea urchin sperm protein, Enterokinase, Agrin (SEA) domain divides the pre-peptide into α and β-subunits (αDG and βDG), which remain non-covalently associated at the plasma membrane (PM). At its intracellular C-terminus, βDG binds to the cytoskeletal linker protein dystrophin, which, in turn, engages filamentous actin, completing a molecular bridge anchoring the cytoskeleton to the ECM. In turn, the actin cytoskeleton is connected to the nuclear periphery by the Linker of Nucleoskeleton and Cytoskeleton (LINC) complex that is anchored in the nuclear membrane. In this way, a direct physical link is established extending from the ECM to the nucleus of the cell.

In addition to its function at the plasma membrane, evidence of a role for βDG in the nucleus is emerging. Members of the DGC within the nucleus were first identified when the dystrophin splice isoform Dp71 was found to be two species; the Dp71f and Dp71d isoforms which localise to the cytoplasm and nucleus, respectively, in HeLa cells [2]. Interestingly, these observations were recapitulated in C2C12 myoblasts [3], and in both cases, immunoprecipitation experiments indicated the existence of an alternative DGC within the nucleus, although its precise functions have remained elusive. The nuclear localisation of DGC components would appear to be mediated by βDG, which contains a nuclear localization signal (NLS) in its intracellular juxtamembrane region between amino acids 776 and 782, thus facilitating nuclear import through recognition by α- and β-importins [4], which is a conventional pathway transiting the nuclear pore complex. Significantly, the non-covalent heterodimeric organisation of dystroglycan is required for autonomous localisation of the β-subunit to the nucleus, for its initial transit through the secretory pathways to the plasma membrane [5,6]. However, it is currently thought that the plasma membrane is not the only source of nuclear βDG. At least to some extent, ezrin binding of βDG enhances its nuclear localisation specifically from a cytoplasmic pool [7]. Regulation of βDG internalisation from the PM is regulated by its phosphorylation at tyrosine 890 ([p]Y890); however, evidence indicates that βDG exists within the nucleus in both phosphorylated and unphosphorylated forms [8]. Further complicating the understanding of the origin of nuclear βDG is the observations that at least some pY890 βDG is ubiquitinated and degraded by the proteasome after internalisation. Indeed, preventing this phosphorylation event or proteasomal degradation both appear to maintain βDG and the DGC at the sarcolemma, where it can maintain membrane integrity in the absence of dystrophin, as in animal models of Duchenne muscular dystrophy (DMD; [9,10]). The mechanisms behind the sorting of βDG pools and their designation for degradation or further trafficking are unknown. Indeed, whilst the role of phosphorylation for internalisation is well-defined [10,11], whether it has a role in nuclear localisation is equivocal. It is notable that numerous studies investigating the translocation of βDG utilised artificial constructs containing just the β-subunit of dystroglycan. Early studies indicated that a non-phosphorylatable (Y890F) point mutation in βDG causes increased nuclear translocation [4], whereas phosphomimetic Y890E mutations cause preferential relocalisation to endocytic compartments, thought to be late endosomes [11]. Without αDG and its N-terminal signal sequence, it is unclear whether these synthetic proteins are processed physiologically, which may lead to the misinterpretation of localisation data. A more recent study directly comparing the effects of Y890F and Y890E within the context of a full-length dystroglycan construct suggests that phosphorylation is a prerequisite for nuclear import [5]. However, this study does not address how both phosphorylated and non-phosphorylated Y890 βDG of endogenous origin can be found in the nucleus [8].

The most reliable antibodies to βDG recognize the very C-terminal region of the intracellular domain, and they work well in immunofluorescence and Western blotting experiments. The existence of multiple immunoreactive fragments migrating to c. 43 kDa (full length), 31 kDa, and 26 kDa are easily resolved by Western blotting; however, immunofluorescence studies cannot distinguish between 43, 31, or 26 kDa βDG species. The full-length and proteolytic species retain a functional NLS, with the smaller fragments being generated by numerous extracellular and juxtamembranous proteases. Cleavage by extracellular matrix metalloproteases (MMPs)-2 and -9 can release a ~31 kDa βDG species which retains its transmembrane domain [12,13,14],and appears to be cancer-associated [15,16,17]. A further cleavage event in the cytoplasmic juxtamembrane region of βDG is mediated by γ-secretase, which releases the C-terminal soluble ~26 kDa cytoplasmic fragment [8]. The functional contribution of βDG fragments to nuclear structure, or whether C-terminal phosphorylation is an exclusive prelude to ubiquitination and degradation is not known. To date, differences in subcellular trafficking of βDG fragments from the plasma membrane has not been probed in detail.

Deficiencies or mutations in DG in humans are rare and give rise to severe debilitating conditions known as the dystroglycanopathies. Primary dystroglycanopathies describe disease states induced by direct *DAG1* mutations. Comparatively, the tertiary and secondary groups arise due to mutations in other genes which affect the metabolic synthesis and handling of glycosyl precursors, or the enzymes required to adduct them to the nascent dystroglycan peptide, respectively. Primary dystroglycanopathies are rarely reported; the total loss of dystroglycan has been described in just one family [18]. The patient investigated displayed severe Walker–Warburg syndrome (WWS) and died after 3.5 months. Other affected siblings also displayed muscle and neurological defects, consistent with WWS, and died within hours or days of birth, often due to respiratory failure. It therefore seems that the absence of, or mutations in human dystroglycan, are often perinatally lethal. Indeed, *DAG1*-ablated mouse embryos die at day E6.5 due to the requirement of dystroglycan in the formation of Reichert’s membrane [19], without which the embryo cannot implant. It is plausible that dystroglycan has paralogous fundamental roles in early human development.

The uniting paradigm in our current aetiological understanding of the dystroglycanopathies is a disruption of dystroglycan–ECM engagement. This is supported by several incidences of point mutations in αDG which typically result in hypoglycosylation and milder symptoms [20,21,22,23]. 

On the other hand, the β-subunit of dystroglycan is largely devoid of glycosyl modifications, and only two primary mutations have been reported to date. Cys669 resides in the ectodomain and its mutation to Phe, found in a patient with muscle–eye–brain (MEB)-like symptoms with multicystic leukodystrophy, was found to disrupt a disulphide bridge with Cys713 which is known to be essential for the cleavage of dystroglycan into α- and β-subunits [24]. Finally, the sole mutation identified in the cytoplasmic domain of βDG is Arg776Cys, in a patient who displayed late-onset muscular dystrophy. Arg776 resides juxtamembranally and is thought to constitute part of the NLS [4,6], though the contribution of this single arginine residue has never been directly tested. 

Overall, the few primary dystroglycanopathies can be allocated to two groups. The first, and most well understood, concerns mutations that affect glycosylation and thus ECM binding. These give rise to the heterogeneous disease states aligned with secondary dystroglycanopathies. Group two may comprise those where αDG is unaffected and presumed to be appropriately engaged with extracellular ligands, while mutations in the β-subunit affect downstream functions in a mutation-site-specific manner. The existence of only two such naturally occurring mutations, together with the knowledge that βDG can localise to the nucleus, raises the possibility that other, non-plasma-membrane-localised functions for βDG exist that contribute to the normal functioning of the cell.

In this study, we use CRISPR/Cas9 to generate human myoblasts lacking DG. We find that multiple clones do not display the previously reported phenotypes attributed to dystroglycan deficiency such as defects in nuclear shape. We further characterise DG-knockout (KO) cells using atomic force microscopy (AFM) and find no significant deviation in stiffness from wild-type (WT) clones; however, the KO of DG in myoblasts appears to result in increased nuclear size, which is an effect rescued upon the re-expression of dystroglycan. To further explore the contribution of βDG to the cell nucleus, we describe the characterisation of epitope-tagged DG constructs which we posit as a valuable tool to indicate that βDG originating from the full-length construct cannot be detected within the nucleus, whereas truncation constructs encoding just βDG distribute as previously reported, but differently to the full-length version.

## 2. Materials and Methods

### 2.1. Cells and Generation of DAG1 KO Myoblasts

KM155 [25] and *DAG1* KO KM155 derivatives were maintained in the skeletal muscle cell growth medium (PromoCell, Heidelberg, Germany) and routinely subcultured by detachment using 0.05% trypsin–EDTA (Gibco, Paisley, Scotland, UK). HeLa and 293T cells were maintained in DMEM supplemented with 10% FBS and 1× GlutaMAX (Gibco, Paisley, Scotland, UK).

For dystroglycan-knockout KM155 myoblasts generation, 2 gRNA sequences with targets near to the *DAG1* start codon (sequence in Supplementary Figure S1A) were cloned into the pSpCas9(BB)-2A-GFP construct (a gift from Feng Zhang; Addgene plasmid #48138). The vectors were co-transfected into KM155 myoblasts and cloned by single-cell sorting. Single-cell clones were grown to 60% confluence, and the plate duplicated for In-Cell Western screening using MANDAG2 antibody staining (see Supplementary Table S1 for a list of antibodies used). MANDAG2-negative clones were further expanded and characterised as described.

### 2.2. Cellular Fractionation

As described in [26], briefly, cells grown to 80% confluency in a T175 flask were washed twice in ice-cold 1× PBS and scraped into 2 mL of ice-cold 1× PBS. Cells were then centrifuged at 3500× *g* at 4 °C for 15 min (Sigma, St. Louis, MO, USA 1–15 K). The pellet was then resuspended in 0.5 mL of fractionation TM buffer (10 mM Tris-HCl pH 8.0, 2 mM MgCl_2_, 0.5 mM PMSF, 1× protease inhibitor cocktail (Roche, Basel, Switzerland)) and incubated on ice for 10 min. A total of 0.5 mL of 2% Triton X-100 (Sigma, St. Louis, MO, USA) in PBS was then added to the cell suspension and incubated on ice for 10 min. The cell suspension was then transferred to a Dounce homogeniser (Wheaton; DWK Life Sciences, Stoke-on-Trent, UK) and the cells were homogenised with between 18 and 26 strokes. The membrane integrity was monitored using an inverted microscope (CETI) at 10× magnification. The cell suspension was then centrifuged at 3500× *g* at 4 °C for 15 min. The supernatant containing the non-nuclear fraction was saved for further analysis. The pellet contained the nuclear fraction and was resuspended in 0.5 mL of fractionation buffer I (0.32 M sucrose, 3 mM CaCl_2_, 0.1 mM EDTA, 10 mM Tris-HCl pH 8.0, 1 mM DTT, 0.5 mM PMSF, and 0.5% NP-40). A total of 0.5 mL of fractionation buffer II (2 M sucrose, 0.1 mM EDTA, 10 mM Tris-HCl pH 8.0, 1 mM DTT, and 0.5 mM PMSF) was then added to the nuclear suspension and pipetted to ensure thorough mixing. In an ultra-centrifuge tube (Beckman-Coulter, Indianapolis, IN, USA), 1 mL of fractionation buffer II was added and the nuclear suspension was laid on top with 3 mL of fractionation buffer I being laid on top of the nuclear suspension. This produced a sucrose gradient which was then centrifuged at 80,000 rpm using the MLA-80 rotor at 4 °C for 1 h. The supernatant was then removed, the pellet was resuspended in 4 mL PBS, this was then added to the poly-L-lysine (Sigma, St. Louis, MO, USA) coated 40 × 11 mm petri dishes (TPP^®^, Trasadingen, Switzerland), and left to attach for at least 30 min. After at least 30 min, the PBS was removed and fresh PBS was added; PBS was then removed and cell growth media was added ready for measurements.

### 2.3. Cell Cycle Synchronisation by Serum Starvation

Furthermore, 24 h prior to AFM analysis, the media on the cells was aspirated, the cells washed in 1× PBS, and the media replaced with skeletal muscle cell growth medium without the supplement addition. The cells were returned to the incubator. After 24 h, the cells were again washed in PBS and the media was replenished with supplemented media, prior to AFM measurement.

### 2.4. SDS-PAGE and Western Blotting

Adherent mammalian cells were lysed in cold RIPA buffer (150 mM NaCl, 1% NP-40 (*v*/*v*), 0.5% sodium deoxycholate (*w*/*v*), 0.1% SDS (*w*/*v*), 25 mM Tris pH 7.4) supplemented with 1× protease inhibitor cocktail (Roche) and 1 mM sodium orthovanadate. Protein samples were run on 10–12.5% hand cast tris-glycine gels and transferred to PVDF membranes by wet transfer. The resulting Western blots were blocked in 5% (*w*/*v*) skimmed milk powder in tris-buffered saline with Tween 20 (TBST; 5 mM Tris-Cl, 15 mM NaCl, 0.2% Tween-20 (*v*/*v*), pH 7.6), and all the antibodies (Supplementary Tables S1 and S2) were diluted into the blocking solution. Blots were developed using SuperSignal West Femto ECL (ThermoFisher Scientific, Waltham, MA, USA) and imaged on either BioRad ChemiDoc, or X-ray film and scanned. 

### 2.5. PCR and Agarose Gel Electrophoresis

All PCR reactions were performed using KOD polymerase (Toyobo, Osaka, Japan). Primers (Supplementary Table S3) were designed to have a melting temperature of 60 °C. Reactions were typically 50 μL and assembled as per the manufacturer’s instructions with 200 μM dNTPs, 2 mM MgSO_4_, 0.3 µM forward and reverse primer, and 2% DMSO. PCR products were analysed by electrophoresis through 1% *w*/*v* agarose gels in 1× Tris-acetate-EDTA buffer.

### 2.6. RNA Extraction and cDNA Generation

RNA was purified from cultured cells using TRIzol reagent. TRIzol reagent was added directly to cultures and incubated for 5 min at room temperature. Chloroform was added at ratio of 1:5 per volume TRIzol used for lysis. The sample was then centrifuged at 12,000× *g* at 4 °C, and the RNA-containing aqueous phase precipitated by adding 0.5× TRIzol lysis volume of isopropanol. Total RNA was pelleted by centrifugation at 12,000× *g* for 10 min at 4 °C. The pellet was washed in ice-cold 75% ethanol and resuspended in Diethyl-pyrocarbonate (DEPC)-treated H_2_O. RNA concentration was determined by Nanodrop for downstream applications. cDNA was produced from RNA using the High-Capacity cDNA Reverse Transcription Kit (Applied Biosystems, Waltham, MA, USA) and the reactions were assembled as per the manufacturer’s instructions.

### 2.7. Molecular Cloning

Human βDG was amplified from cDNA using KOD polymerase (Toyobo, Osaka, Japan) using primers with restriction sites compatible with the pcDNA3.1 target vector. PCR products and target vectors were then digested using compatible restriction enzymes (NEB) and ligated using T4 ligase (NEB), as per the manufacturer’s instructions. Epitope tags were inserted through the DG cDNA using the overlap extension PCR reaction (Supplementary Table S4), whereby the epitope tags were encoded into the primer oligonucleotides as an overhang. Ligated products were transformed and propagated in TOP10 chemically competent bacteria.

### 2.8. TOPO-TA Cloning for DAG1 KO Characterisation

Genomic DNA was extracted from putative *DAG1*KO clones using QuickExtract lysis buffer (Biosearch Technologies, Hoddesdon, UK). The Cas9-targeted region was amplified using KOD polymerase. After gel extraction, PCR products were 3′ adenylated by incubating the gel-purified KOD PCR product with Taq-containing MangoMix (Bioline, London, UK) at 72 °C for 30 min. PCR products were PCR purified (Qiagen, Hilden, Germany) and ligated into the TOPO-TA vector (ThermoFisher Scientific, Waltham, MA, USA). Then, the ligated products were transformed into bacteria. At least 10 TOPO-TA colonies from each *DAG1*KO clone were Sanger sequenced to determine zygosity.

### 2.9. Plasmid Transfection Procedures

Cells were evenly plated 24 h prior to transfection, such that they would be 70–90% upon transfection. The next day, the cells were transfected using Lipofectamine 3000 according to the manufacturer’s instructions. Lipofectamine-containing media was changed after 6 h. Cells were harvested or fixed for staining 18 h after transfection.

### 2.10. Immunofluorescence Staining and Microscopy

Samples were fixed in 3.7% (*v*/*v*) paraformaldehyde (PFA; Sigma, St. Louis, MO, USA) and incubated at room temperature for 10 min. Cells were permeabilised using 0.2% Triton X-100 (Sigma, St. Louis, MO, USA) in PBS and incubated at room temperature for 3 min. All primary and secondary antibodies were diluted in blocking buffer (3% BSA (*w*/*v*), 5% FBS (*w*/*v*) in PBS). Antibody dilutions can be found in Supplementary Tables S1 and S2. Samples were counterstained with DAPI (Sigma, St. Louis, MO, USA) at 10 ng/mL and mounted in either ibidi mounting medium (ibidi) or hydromount (National Diagnostics) containing 2.5% 1,4-Diazabicyclo [2.2.2] octane (DABCO; Sigma, St. Louis, MO, USA).

Widefield fluorescence images were acquired on a DMIRE2 inverted fluorescent microscope which was controlled by a Leica CTRMIC controller. Leica filters A4 (DAPI; excitation at 360 nm and emission at 400 nm), N2.1 (TexasRed, AlexaFluor 594; excitation 515–560 nm and emission at 580 nm), and L5 (GFP, FITC, AlexaFluor 488; excitation at 480 nm and emission at 505 nm) were used. A Leica DC350F CCD camera was used and the images were acquired using the Q-Fluoro software version 1.2.1, (Leica Microsystems, Cambridge, UK).

Confocal imaging was performed either using a Nikon A1 equipped with a CFI Plan Apochromat VC 60× oil (NA 1.4) objective lens (Wolfson Microscopy Facility, Sheffield, UK) or an Olympus FV3000RS (A*STAR Microscopy Platform, Singapore) equipped with a UPlanSApochromat 60× oil (NA1.35) objective. Optical sections were maximally projected for intensity quantification. A Z-step of 0.125 μm was used for nuclear volume analysis.

### 2.11. Atomic Force Microscopy

Tipless MLCT-O10 AFM probes (Bruker, Billerica, MA, USA) were used (spring constant between 0.020 and 0.025 N/m, length between 195 and 205 μm, width between 15 and 25 μm). The cantilever was then mounted on the AFM (Nanowizard III, JPK Instruments, Berlin, Germany). Then, 5 μm diameter polystyrene spheres (Sigma, St. Louis, MO, USA) were attached to the tipless cantilever using UV-curing adhesive (Norland Optical Adhesive 81, Norland, Jamesburg, NJ, USA) which was cured using UV light for at least 5 min.

Data were acquired using the JPK Nanowizard III with either modified tipless MLCT-010 with a nominal spring constant of 0.02 N/m or MLCT-SPH-5UM (Bruker, Billerica, MA, USA) with a nominal spring constant of 0.03 N/m. The JPK Nanowizard was mounted on a Nikon A1 inverted microscope. Prior to measurements, the cantilever was calibrated using the thermal vibration method to determine the spring constant. Prior to measuring the cells, the sensitivity of the cantilever in the cell growth media at 37 °C was determined using the contact-based method. Using a 40× magnification on the optical microscope, a single cell was identified and either the area over the nucleus or an area of the cytoplasm was measured with an approach speed of 5 μm/s and a setpoint of 3 nN. Each area of the sample was measured at least 10 times to give an average of each sample point and allowed the removal of unsuitable curves while maintaining multiple usable curves for each sample point. For each experiment, at least 15 cells were measured for each sample. For the isolated nuclei, functionalised MLCT-O10 cantilevers were used with an extend speed of 3 μm/s and a setpoint of 1 nN. For each experiment, at least 10 nuclei were measured for each sample.

### 2.12. Live Cell Imaging for Transwell Assays

Live cell images were acquired using the inverted Ti eclipse Nikon Widefield system equipped with a Plan Apo 20× (NA 0.75) objective, SpectraX LED excitation (395 nm, 440 nm, 470 nm, 508 nm, 561 nm, 640 nm), Quad filter for DAPI/GFP/RFP/Cy5, and Andor Zyla sCMOS camera (2560 × 2160; 6.5 μm pixels). The environmental conditions of 37 °C with 5% CO_2_ were maintained using the Oko-lab environmental control chamber. Image acquisition used the NIS Elements Software Version 5.21.00.

Control and *DAG1* KO myoblasts were stained with CellTrackerTM Red CMTPX (Invitrogen, Waltham, MA, USA) and CellTrackerTM Green CMFDA (Invitrogen, Waltham, MA, USA), respectively. Control and KO cells were then co-cultured in a glass bottom petri dish (Ibidi, Fitchburg, WI, USA). Each experiment was imaged over 15 h with each XY position imaged every 10 min. For each time point, a brightfield image was taken in addition to images in the red and green channels to identify the two cell types.

### 2.13. Image Analysis

#### 2.13.1. Nuclear Morphology

Nuclear shape descriptors were quantified on CellProfiler version 3.1.9 or FIJI version 1.0. Briefly, nuclei, as demarcated using DAPI, were identified using the IdentifyPrimaryObject module, and the form factor was calculated using the MeasureCellSizeShape module. Data were exported into GraphPad Prism version 8.0, files for statistical analysis. Nuclear area was quantified using FIJI. Briefly, the images were manually thresholded to include the entire area and area quantified using the analyse particles command.

#### 2.13.2. Cell Migration Analyses

Generated time lapse images were processed using FIJI using the Manual Tracking plugin and the Chemotaxis and Migration Tool 2.0 (Ibidi, Fitchburg, WI, USA) plugin. Each XY position was separated into an individual time lapse image sequence and then the three channels were split. The cells were manually tracked and this data was then entered into the Chemotaxis and Migration Tool which then calculated migration velocity and total migration distance. This data was then exported to Excel to collate and organise it and then imported into GraphPad Prism for statistical analysis.

## 3. Results

### 3.1. Generation of Dystroglycan-Knockout Human Myoblasts Using CRISPR/Cas9

**Figure 1:**
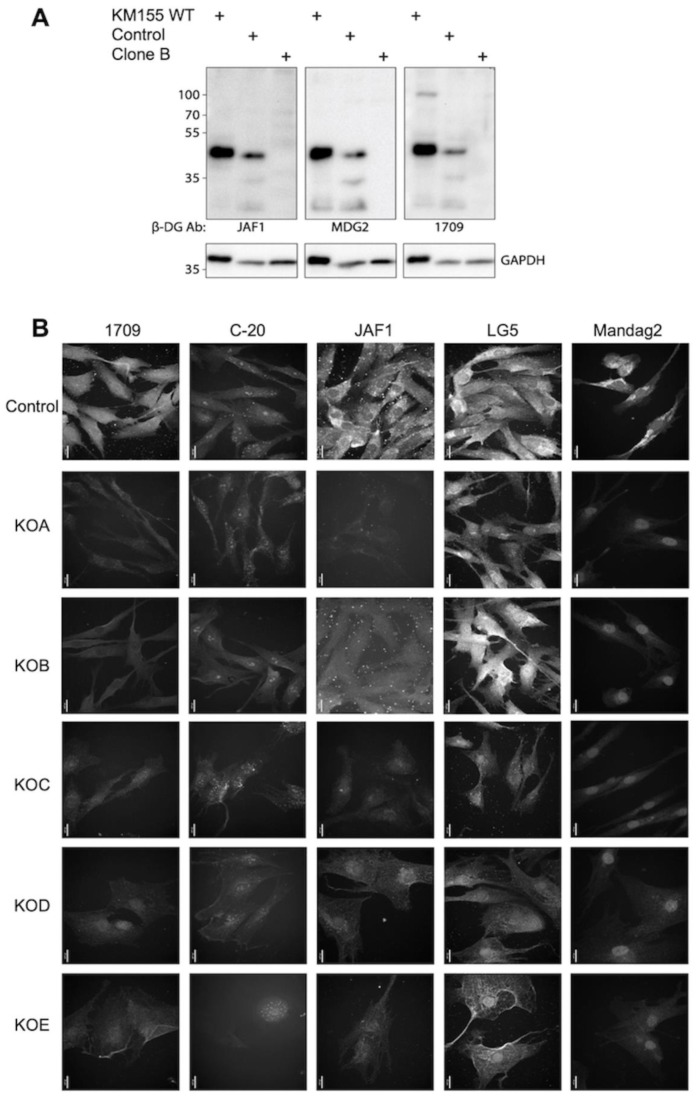

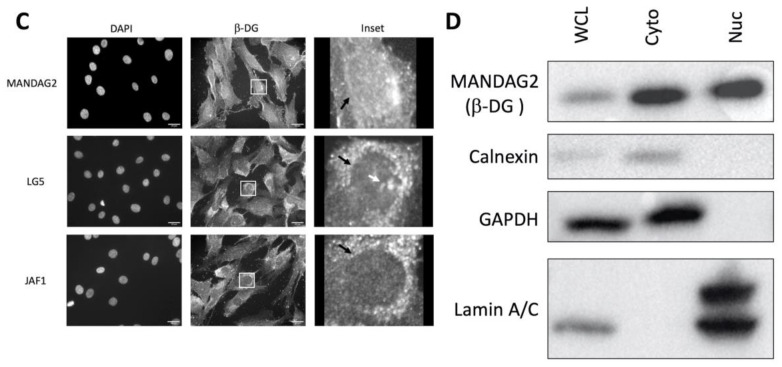
Characterisation of βDG antibodies in KM155 myoblasts. (**A**) Characterisation of MANDAG2, JAF1, and 1709 in WT and *DAG1* KO KM155 myoblasts by Western blotting. (**B**) WT and *DAG1* KO KM155 myoblasts were stained with βDG antibodies MANDAG2, JAF1, C-20, LG5, and 1709 to characterise antibody specificity. Scale bar = 20 μm. (**C**) βDG is detected in the nuclear substructures (black arrows) using MANDAG2, JAF1, and 1709. Inset boxes represent location of enlarged images at right. Arrows indicate βDG localisation at the nuclear envelope. Scale bar = 20 μm. (**D**) Cellular fragmentation of KM155 myoblasts demonstrates the presence of βDG in the nuclear fraction, as detected using MANDAG2.

To investigate the role of dystroglycan in the nucleus, we generated a human myoblast line with dystroglycan knockout by CRISPR/Cas9. KM155 normal human myoblasts were acquired from [25] and co-transfected with two Cas9-GFP vectors harbouring gRNAs targeting either side of the *DAG1* start codon (Figure 1A). After transfection, GFP+ cells were single-cell sorted and incubated for clonal expansion. Clones were screened for *DAG1* KO by In-Cell Western, and promising clones were further analysed by Western blotting and Sanger sequencing to establish the true knockout clones (Supplementary Figure S1B,C). The clones generated can be seen in Table 1, with those used in this study highlighted. 

### 3.2. Establishing Specific Dystroglycan Antibodies and Localisation to the Nucleus

Numerous antibodies are available for dystroglycan; however, many have not been KO tested. The availability of human *DAG1* KO myoblasts permitted the screening of available antibodies by Western blotting and immunofluorescence to establish those which are fit for purpose. MANDAG2, 1709, and JAF1 detected full-length βDG at 43 kDa and a number of fragmentation products in WT and CRISPR control cells. These bands were not detected in *DAG1* KO cells (Figure 1A). Using immunofluorescence, it was found that C20 and LG5 staining was not reduced in *DAG1* KO clones, indicating non-specific staining (Figure 1B). It should also be noted that other *DAG1* antibodies, MANDAG2, 1709, JAF1, displayed a level of background staining which should be accounted for during analysis.

All βDG antibodies trialled detected the extreme C-terminus of the protein, a region thought to be retained in all fragmentation products, meaning localisation observations are difficult to interpret. As previously observed, staining KM155 cells with MANDAG2, 1709, or JAF1 revealed non-discrete βDG fluorescence in the nuclear region (Figure 1C). Cellular fractionation and SDS-PAGE analysis of nuclear samples appeared to confirm these observations (Figure 1D).

### 3.3. Generation of an Epitope-Tagged Version of DAG1 to Establish Subcellular Compartmentalisation

**Figure 2:**
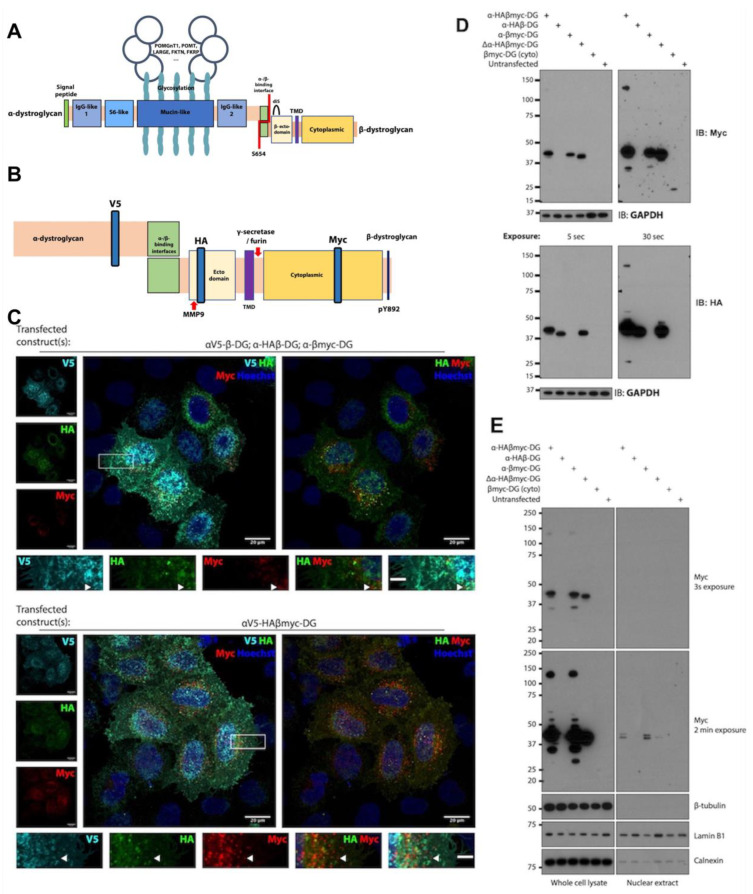
Multiple epitope tags in dystroglycan reveal the spatial resolution of the β-subunit fragments. (**A**) Schematic diagram of the mature *DAG1* protein, cleaved into α- and β-subunits. (**B**) Epitope tag placement in the carboxy-terminus of αDG and throughout βDG. (**C**) Epitope tags within the αV5-HAβmyc dystroglycan construct become separated upon its expression in HeLa cells. HeLa cells were either triply transfected with a version of *DAG1* modified by a single epitope tag (**upper**), or with the αV5-HAβmyc construct (**lower**). Transfected HeLa cells were probed using antibodies specific to the epitope tags through the dystroglycan construct to reveal the localisation of fragments relative to one another. Boxed region shown enlarged in lower panels. Arrowheads denote regions which are myc stained but not HA. Scale bar full image = 20 μm. Scale bar inset = 5 μm. (**D**) βDG fragments differently when expressed in the absence of α-dystroglycan. HeLa cells were transfected with the indicated construct, and βDG fragments arising from each construct were analysed by Western blotting. Upper panels show myc immunoreactive bands, while lower panels show HA. Multiple exposures of Western blots reveal less abundant fragments. (**E**) HeLa cells transfected with the indicated constructs were fractionated to isolate nuclei. Whole-cell lysates and nuclear extracts were then analysed by Western blotting and probed with antibodies raised against the myc epitope within the construct. The presence of lamin B1 and absence of β-tubulin was used to verify the enrichment of nuclei, while calnexin was used as a measure of purity. … denotes a non-exhaustive list of the enzymes involved in the glycosylation of dystroglycan.

To circumvent ambivalence in the antibody detection of endogenous dystroglycan, and to generate a versatile tool to probe dystroglycan fragment function, we devised an epitope-tagging strategy whereby previously identified DG fragmentation products were labelled with unique antibody-recognisable labels (Figure 2A,B). To validate the system, the triple-tagged construct was transfected into HeLa cells while constructs containing each of the tags separately were co-transfected as a positive control. As expected, we observed the myc-tag, marking the cytoplasmic fragment, spatially separated from the HA-tag by immunofluorescence microscopy, indicating functional proteolytic cleavage in the multi-epitope modified construct (Figure 2C). Interrogation of triple-tagged constructs by SDS-PAGE revealed that the V5-tag in αDG partially inhibited its normal proteolytic cleavage (Supplementary Figure S2A). Given our interest in the subcellular function of αDG, we removed the V5-tag. The analysis of cells transfected with α-HAβmyc-DG by SDS-PAGE revealed multiple products derived from the full-length constructs, most notably a myc-only immunoreactive band at c. 30 kDa, which is consistent with that observed from the endogenous protein (Figure 2D). Interestingly, expression of a βDG construct, in the absence of αDG, did not yield the lower MW myc-only immunoreactive band (Figure 2D). In fact, both βDG and βDGcyto truncations of DG both yielded different subcellular localisations to one another and were not reminiscent of the full-length construct (Supplementary Figure S2B). This finding suggests that DG fragment trafficking and localisation is largely determined by its normal endogenous context.

### 3.4. DAG1 KO Cells Do Not Display a Defect in Nuclear Morphology

Defects in nuclear morphology have been reported in a number of muscular dystrophies of unclear aetiology [27]. To probe whether *DAG1* plays a role in nuclear stability, we next analysed multiple clones of *DAG1* KO myoblasts for nuclear phenotypes which may indicate a function for DG in the nucleus.

**Figure 3:**
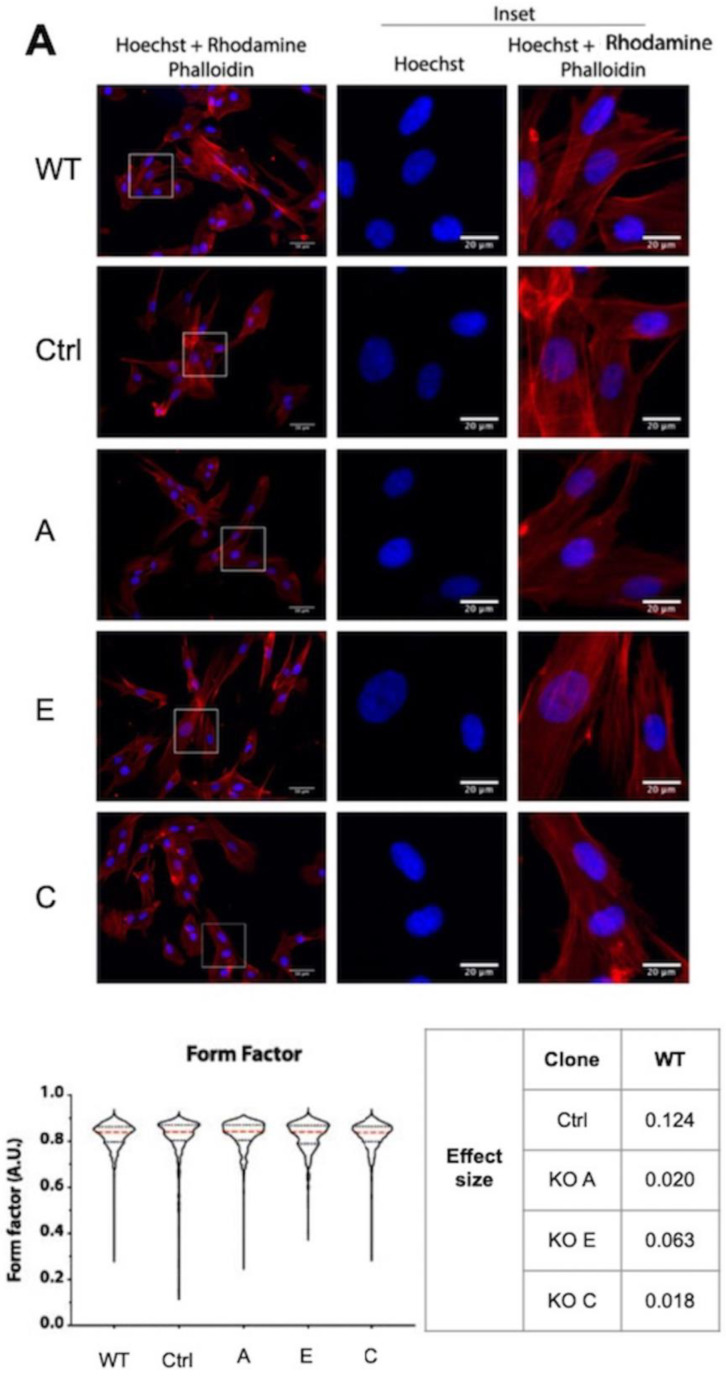

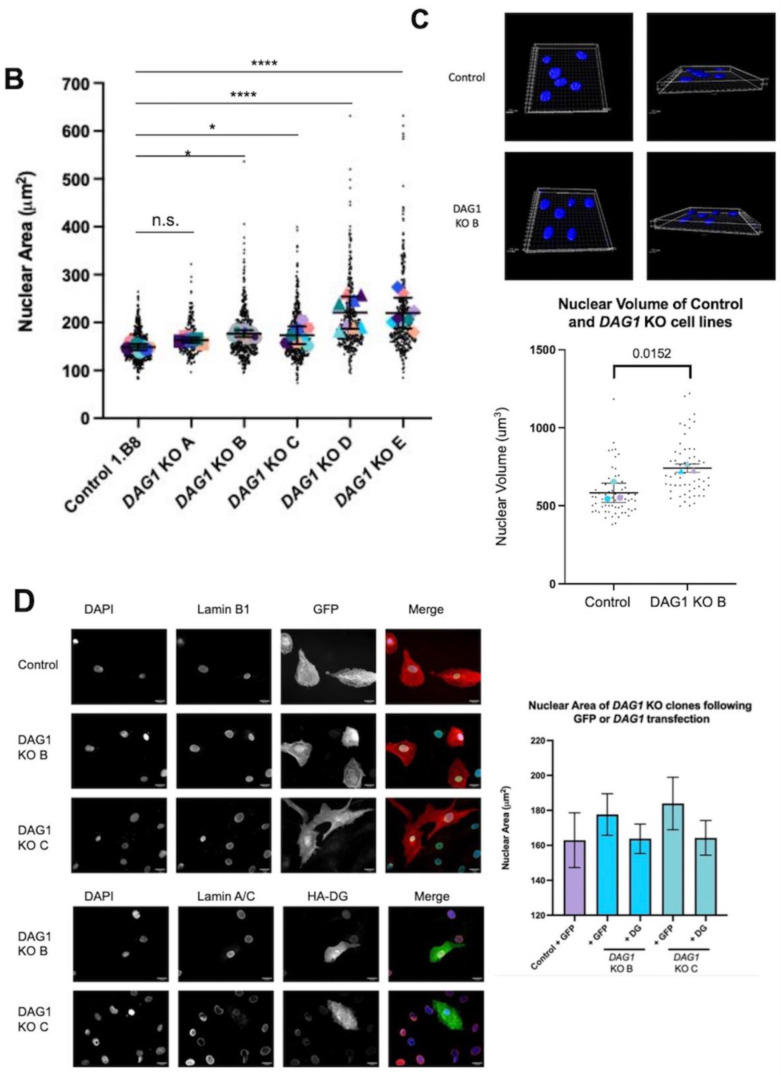
Loss of dystroglycan increases nuclear size but not gross morphology. (**A**) WT and *DAG1* KO KM155 cells were fixed and stained with DAPI and phalloidin-AF568 and nuclear shape was quantified using the cell profiler. Scale bars = 20 μm. Form factor shown and differences presented as effect size compared to WT. Magnitude of shift, indicative of biological relevance, was compared against the established categories for effect size (very small, 0.01; small, 0.2; medium, 0.5; large, 0.8; very large, 1.2) [29,30]. Data from 3 independent experiments, except clone E which became senescent, comprising 119–373 cells for each condition. Tested for normality using D’Agostino and Pearson’s normality test followed by Kruskal–Wallis test with Dunn’s multiple comparisons. Significance is reported where effect size is >0.2 (small). (**B**) WT and *DAG1* KO KM155 myoblasts were fixed and stained with DAPI and nuclear area was quantified. Data from 7 independent experiments, at least 738 nuclei from each condition quantified. Small black points indicate values from individual cells while larger coloured points indicate the average for each independent experiment. One-way ANOVA with Fisher’s least significant test for multiple comparisons. * *p* ≤ 0.05; **** *p* ≤ 0.0001. n.s. not significant. (**C**) Nuclear volume—cells were grown for 24 h before fixing and staining with DAPI to identify the nuclear region. Cells were imaged using a Nikon A1 confocal microscope and the 3D nuclear volume determined for analysis. (upper) Representative reconstructions using Imaris software version 9.2. Graph showing nuclear volume of control and *DAG1* KO clone B. Small black points indicate values from individual cells while larger coloured points indicate the average value for each independent experiment. A total of 3 independent experiments were carried out with 45 control and 49 KO B nuclei measured. Graph shows mean and standard deviation. A significant difference was determined between the control and *DAG1* KO B as determined by the *t* test, *p* = 0.0152. (**D**) *DAG1* KO clones B and C were transfected with either GFP or α-HAβmyc-DG constructs, fixed and stained for HA. Nuclear area was quantified in the transfected cells based on HA staining or GFP positivity. Scale bar = 20 μm. Magnitude of difference is displayed as effect size, compared to WT nuclear area measurement.

We initially quantified the nuclear morphology in *DAG1* KO myoblasts which is indicative of alterations to nuclear mechanics [28]. No significant differences were observed in shape descriptors such as form factor in multiple *DAG1* KO clones (Figure 3A). However, the nuclear area appeared to be increased in the absence of *DAG1* (Figure 3B). Consistent with the increase in nuclear size, upon confocal analysis of *DAG1* KO clone B, we found that the volume was also increased (Figure 3C). The increase in nuclear area was robustly rescued and returned to WT size by the re-expression of the epitope-tagged DG construct (Figure 3D).

**Figure 4:**
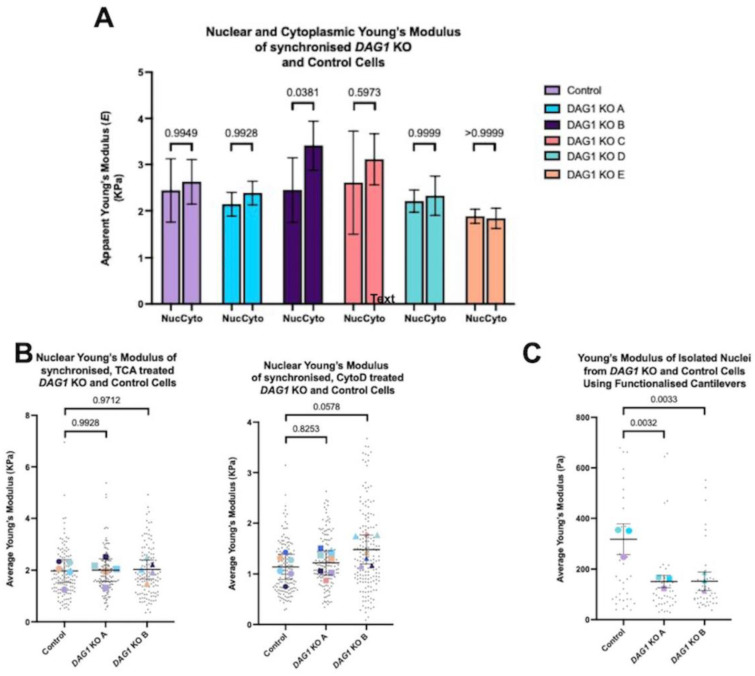
Nuclear atomic force microscopy in myoblasts deficient in *DAG1*. (**A**) Nuclear and cytoplasmic Young’s Modulus of G1 synchronised WT and *DAG1* KO KM155 cells. Cells were measured using commercial cantilevers. A total of 6 independent experiments were carried out with 90 individual cells measured per sample. Graph shows mean and standard deviation; *p* values for multiple comparisons using Šídák’s multiple comparisons test are in the graph. (**B**) Nuclear Young’s Modulus of WT and *DAG1* KO KM155 cells following treatment with 300 nM TCA or 500 nM Cytochalasin D. Nuclei were measured using a functionalised cantilever. Small black points indicate values from individual cells while larger coloured points indicate the average for each independent experiment. A total of 5 independent experiments were carried out with 100 individual nuclei measured per sample. Graph shows mean and standard deviation. A significant difference was not found between any of the samples as determined by the one-way ANOVA test *p* = 0.9764. The *p* values for multiple comparisons using Dunnett’s multiple comparisons test are in the graph. (**C**) Young’s Modulus of the isolated nuclei from WT and *DAG1* KO KM155 cells. Mechanical properties of isolated nuclei from control and *DAG1* KO A and B as measured by a functionalised MLCT cantilever. Small black points indicate individual nuclei while larger coloured points indicate the average of each experiment. Two individual outlier points from the control column were excluded to enable better visualisation; these values were 1121 Pa and 2553 Pa. *n* = 3 independent experiments with 35 individual cells measured per sample (except control where *n* = 36). Statistical analysis using one-way ANOVA test *p* = 0.0047. *p* values for multiple comparisons using Dunnett’s multiple comparisons test are in the graph.

We reasoned that this dilation of nuclear size may be a function of nucleoskeletal weakness induced by the loss of *DAG1*. To explore nuclear stiffness, we subjected multiple *DAG1* KO myoblast clones to atomic force microscopy (AFM) to generate absolute values for the Young’s modulus in the presence and absence of *DAG1*. To ensure the stiffness measurements were not affected by cell cycle, we synchronised the cells by serum starvation and validated G1 arrest by flow cytometry. Unexpectedly, we observed no difference in stiffness over the nuclear area or the cytoplasmic region in multiple synchronous *DAG1* KO myoblasts clones upon G1 cell cycle synchronisation (Figure 4A). In order to isolate the mechanical contributions of chromatin or the actin cytoskeleton, the cells were treated either with TCA or cytochalasin D (CytoD) to induce chromatin decondensation or actin cytoskeleton depolymerisation, respectively, before nuclear AFM probing. As in the untreated conditions, we found that the absence of dystroglycan had no effect on nuclear stiffness upon treatment with either TCA or CytoD (Figure 4B).

To exclude the contribution of any other endogenous cellular factor to the measured nuclear area stiffness, we refined a nuclear isolation protocol to probe purified isolated nuclei by AFM. The final procedure was validated by immunofluorescence microscopy demonstrating the absence of residual endoplasmic reticulum in the nuclear preparation (Supplementary Figure S3). In contrast to all the experiments performed in intact cells with or without treatment, the AFM-determined Young’s moduli of the isolated nuclei indicate that the absence of dystroglycan results in decreased nuclear stiffness in both clones tested (Figure 4C). The observation that isolated nuclei deficient in dystroglycan are softer than controls, but not in whole cells treated with either TCA or CytoD indicates that the microtubule or intermediate filament cytoskeleton has a greater effect on the perceived nuclear integrity than anticipated. Alternatively, there may be compensatory mechanisms in *DAG1* KO cells that mask the phenotype in a whole cell context. Complementation of epitope-tagged dystroglycan in *DAG1* KO cells to demonstrate the DG specificity of this observation was complicated by the low transfection efficiency of human myoblasts.

### 3.5. DAG1 KO Nuclei Are Not More Sensitive to Mechanical Strain than WT Cells

Many genes associated with MDs produce structural proteins. Myofibres are thought to be particularly sensitive to the germline loss of these genes due to their dependence on structural proteins to resist and protect the cell from strain induced by the tissue [31,32]. We therefore sought to apply strain to the *DAG1* KO myoblasts with the hypothesis that a nuclear phenotype may be induced under stressed conditions. To simulate compressional forces which cells undergo in stressed tissues such as muscle, myoblasts were seeded into transwells with 3 μm pores which cells could migrate through. As a proof of concept that the engineered KO cells are functionally *DAG1* defective, we measured migration speed compared with the control cells. We found that *DAG1* KO myoblasts reproducibly migrated at reduced velocity compared with WT cells in fitting with the function for plasma membrane dystroglycan in cell adhesion and migration [33] (Supplementary Figure S5). However, quantification of the size and shape of the nuclei after migration through the 3 μm transwell pores revealed no significant difference upon the loss of dystroglycan.

**Figure 5:**
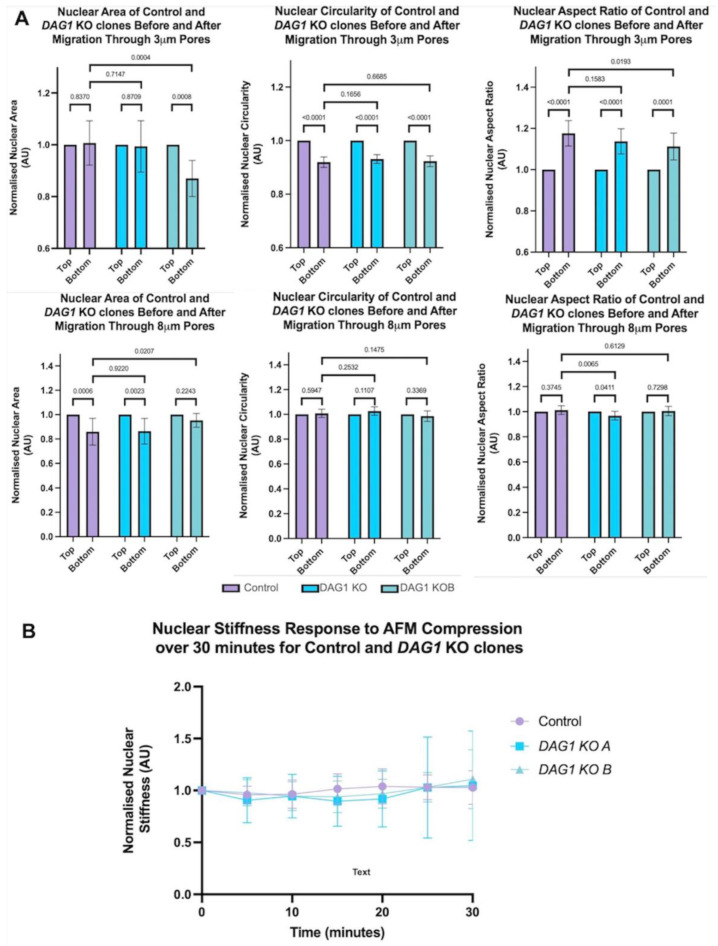
Myoblasts lacking *DAG1* do not display hypersensitivity to mechanical strain. (**A**) Nuclear size and shape quantification after migration through 8 μm or 3 μm transwell pores. Nuclear morphology was measured in cells which had and had not migrated through the transwell pores. Nuclear morphology parameters are displayed on graphs. At least 150 cells were measured for each condition. One-way ANOVA with Šídák’s multiple comparisons test. (**B**) Nuclear response over time to repeated presssure by AFM in WT and *DAG1* KO nuclei. A total of 1 kPa was applied to the nuclei and nuclear stiffness was measured every 5 min for a total of 30 min.

In contrast to migration through an 8 μm control pore which resulted in decreased nuclear size in the control and *DAG1* KO clones alike, migration through the 3 μm pores did not significantly affect the nuclear size of the control cells (Figure 5A). Notably, one of the two tested *DAG1* KO clones retained nuclear size following migration through the 3 μm pores and one clone had a significant reduction in nuclear size following migration (Figure 5A). Unlike migration through the 8 μm control pores, migration through the 3 μm pores induced severe defects in nuclear morphology after migration to the bottom of the transwell, as measured by form factor. However, the absence of dystroglycan did not further exacerbate this feature.

Various studies have indicated that nuclei can adapt their mechanical properties in response to consistent or repeated stressors [34]. We therefore sought to determine whether KO of *DAG1* modified the stiffness of nuclei over time compared with WT cells. A repeated pressure of 1 kPa, a pressure in the physiological range previously applied to muscle cells [35], was applied by AFM and measurements were made every 5 min. Over the 30 min experiment, we found that the degree of nuclear stiffness in the WT cells changed markedly, but the absence of dystroglycan appeared not to modify this (Figure 5B) and there were no statistically significant differences between the wild-type and KO clones.

## 4. Discussion

Dystroglycan, within the dystrophin-associated glycoprotein complex has a well-established role in the maintenanceof myofibre sarcolemmal stability and as a signalling complex [1,36]. Loss of, or mutation in, dystroglycan or enzymes required for its appropriate post-translational modification result in various muscle wasting diseases [37,38]. Increasing numbers of publications have detected dystroglycan in the nucleus of various cell types. Even though nuclear abnormalities have been described in the dystroglycanopathies [37], the significance and peptide nature of the nuclear localisation of dystroglycan in skeletal muscle tissue has remained elusive. Given the complexities of known dystroglycan function to date, here we devised tools to study dystroglycan trafficking through engineering epitope-tagged constructs and dystroglycan-knockout human myoblasts for phenotypic analysis. While the loss of dystroglycan from myoblasts did not cause overt nuclear morphological defects, nuclear size was increased and reverted to normal upon the re-expression of full-length dystroglycan.

Given the myriad roles already ascribed to βDG and its fragments, we posit that epitope tagging dystroglycan may be a powerful strategy in separating its roles. The clear separation of the myc- from the HA-epitope is indicative that myoblasts have mechanisms to distinguish fragments from one another. However, the importance of careful DG modification and robust characterisation is exemplified by our observation that the insertion of a V5 tag after F613 reduced the autoproteolysis of the dystroglycan pre-peptide. The observation that modifications around the SEA domain impede α/β autoproteolysis correlates well with previous studies where even distal mutations affected cleavage kinetics. Indeed, failure to cleave αDG from βDG can cause MEB disease [6,39,40,41]. 

A striking observation made during the course of this study was the clear difference in the subcellular localisation of truncated dystroglycan constructs. The expression of full-length tagged dystroglycan was initially hypothesised to localise in such a way that represented a sum of the individual dystroglycan fragments. Expression of βDG alone did not demonstrate comparable subcellular localisation while fragmentation was not observed, indicating the necessity of the α-subunit for processing. Interestingly, however, the determined localisation of the truncated βDG constructs in this study was not unprecedented. The expression of full-length βDG has been used extensively to study nuclear functions and is routinely observed in an ER-like distribution [4,11,26,42,43]. Confocal analysis of C2C12 myoblasts expressing full-length βDG also indicates its presence throughout the nucleus, though how it escapes the membranous environment is unclear [43]. Consistent with results from this study, research focussed on the cytoplasmic region of dystroglycan report robust nuclear accumulation [6,16,44,45], though whether this localisation is relevant in the full-length context is unclear.

Notably, despite the robust nuclear localisation of the cytoplasmic tail βmyc-DG to the nucleus, we did not detect a significant accumulation of dystroglycan derived from the full-length construct within the nucleus by immunofluorescence analyses.

The results from the Western blotting experiments of DG constructs were ambiguous. The absence of β-tubulin from the nuclear fraction would indicate that the βDG detected was indeed in the nucleus; however, a more rigorous interrogation of the sample revealed ER contamination due to the presence of calnexin. In fact, the absence of detectable βDG is somewhat in keeping with previous experiments which use full-length dystroglycan constructs. A C-terminal GFP fusion appears to localise to the cytoplasm in REF52 cells and fibroblasts, though epifluorescence microscopy and lack of nuclear counterstain makes this conclusion uncertain [46]. However, the same construct localises exclusively to the cytoplasm in C2C12 myoblasts, unless a Y890E phosphomimetic mutation is introduced leading to an additional diffuse nuclear signal [5].

The rarity of primary dystroglycanopathies indicates the importance of dystroglycan for organismal development and viability. Myofibres and fibroblasts from the very few patients found with disruptions to the dystroglycan locus have been reported to exhibit nuclear abnormalities [18,37,47]. Perturbed nuclear morphologies have also been observed in mouse models of the dystroglycanopathies, although the function of dystroglycan in this regard has not been thoroughly investigated [48]. More recent cell-based studies suggest that dystroglycan functions to maintain nuclear integrity by binding elements of the NL, maintaining the integrity of the NL for normal shape, and nuclear-centrosome anchorage [26,43]. Despite previous compelling data, many aspects of nuclear morphology tested in our *DAG1* KO myoblast model were *DAG1* independent.

Despite the finding that the gross nuclear morphology was unaltered upon the loss of dystroglycan, we did observe a robust increase in nuclear size. Increased nuclear size was rescued upon the re-expression of epitope-tagged DG, indicating the specificity of the phenotype to DG.

Unlike nuclear shape, the nuclear size of cells lacking DG has not been investigated until recently. The only report of nuclear size differences in cells with perturbed DG analyses its association with cellular senescence [49]. This study found that the nuclei of C2C12 mouse myoblasts where *DAG1* had been knocked out had a significantly greater area than WT nuclei. In addition, it also found that the KO cells had a greater area when compared to WT [49]. This is in agreement with the data presented in this study where *DAG1* KO human myoblasts have increased nuclear area.

Despite the previous association of DG with senescence and therefore dilated nuclei [49], our data does not indicate that *DAG1* KO cells have increased rates of senescence. Lamin B1 level alterations, decreased levels of H3K9me3 demarking heterochromatin, or increased DNA damage are all hallmarks of senescence [50,51,52,53,54,55,56]. Testing *DAG1* KO clones A-C for these markers, however, were not consistent with a senescent state (Supplementary Figure S3A,B). We found no reduction in the proliferation rate of KO cells, no difference in the relative levels of heterochromatin marker H3K9me3, the relative levels of γH2AX which is a marker of DNA damage, or the relative levels of lamin B1. An additional argument against a senescent phenotype in *DAG1* KO clones A-C is the evidence of the reversible nuclear size phenotype. Rescue experiments of *DAG1* KO clone B and C using tagged DG resulted in the rescue of the nuclear size phenotype. Given that cellular senescence is defined as irreversible cell cycle arrest, if a lack of DG resulted in true cellular senescence, this should not be rescued by the addition of exogenous DG.

Nuclear stiffness has not previously been investigated in the context of dystroglycan-null cells. The data indicating a reduction in nuclear stiffness when the nuclei are removed from the cellular environment suggests that βDG has a small effect on nuclear stiffness. However, the mean difference between the control and DG KO nuclei was approximately 100 Pa whereas the overall nuclear stiffness for intact cells was approximately 3 kPa and 1 kPa for nuclei in cells with disrupted actin cytoskeletons. Therefore, a difference of 100 Pa within the context of whole cells is unlikely to be of physiological relevance. However, the result does hint at the possibility that βDG is having an as yet undefined role in the nucleus.

## 5. Conclusions

βDG does make a small contribution to nuclear stiffness in myoblasts; how significant this would be in the context of a repeatedly contracting and relaxing muscle fibre is hard to judge with the available data. Loss of βDG leads to significant changes in nuclear area and volume; however, these are completely reversed upon re-expression of dystroglycan. This would tend to suggest a mechanism associated with relaxation in chromatin structure, chromosome tethering/mobility, or ‘loosening’ of the nuclear lamina, rather than an increase in DNA quantity or ploidy. Further experimentation is required to address the mechanisms underlying these changes.

## Figures and Tables

**Table 1:** Generated *DAG1* KO KM155 cell lines and reference in text. Clones were characterised by genotyping PCR, Western blotting, and Sanger sequencing; the results for each are detailed. * denotes a predicted stop codon after amino acid 79.

Clone	PCR Product (bp)	β-Dystroglycan Protein	Mutation Induced	
			gRNA_001	gRNA_002
WT	~840	Expressed	N/A	
10D	~840	Expressed	N/A	
1.B8	~840	Expressed	N/A	
1.B9	~840	Expressed	N/A	
11A	3 bands	Not detected	1 bp deletion	5 bp deletion
1.B6	~630	Not detected	Excision: CT insertion	
1.C2	~630	Not detected	Excision: C insertion	
1.G4	~840	Not detected, predicted aa79 *	1 bp deletion	1 bp deletion
1.G7	~630	Not detected	Excision: C insertion	
2.C7	~630	Not detected	Excision: C insertion	
2.C9	~630	Not detected	Excision: C insertion	
2.D8	~630	Not detected	Heterozygous excision: C insertion and end joining.	

## Data Availability

The original contributions presented in the study are included in the article/supplementary material, further inquiries can be directed to the corresponding author/s.

## Supplementary Materials

The following supporting information can be downloaded at: https://www.mdpi.com/article/10.3390/cells13050431/s1, Figure S1: Generating *DAG1* KO KM155 myoblasts using CRISPR/Cas9; Figure S2: αDG V5 tag placement impedes autoproteolysis; Figure S3: Validation of nuclear isolation; Figure S4: *DAG1* KO human myoblasts appear not to be senescent; Figure S5: Migration velocity and migration distance of *DAG1* KO myoblasts; Table S1: Primary antibodies used; Table S2: Secondary antibodies used; Table S3: Primers used; Table S4: Plasmids used/generated. References [57,58,59,60] are cited in Supplementary Materials.
