# Supplementary material for: The Role of β-Dystroglycan in Nuclear Dynamics
Source: Cells. 2024 Feb 29;13(5):431. doi: 10.3390/cells13050431 (PMC10931191; doi:10.3390/cells13050431)
Supplement: Supplementary file 1 [file cells-13-00431-s001.zip › cells-2788355-supplementary.pdf]

cells

Supplementary Table S1. List of primary antibodies used

Antibody name / target	Antigen	Host species	Isotype	Clone	Dilution WB	Dilution IF	Source
MANDAG2	Ct βDG	Mouse	IgG1	MANDAG2	500	100	[58]
1709	P892 βDG	Rabbit			500	200	[57]
C20		Goat				250	SCBT
LG5	Ct βDG	Rabbit			500	500	D. Mornet lab
JAF1	Ct βDG	Rabbit			500	500	[60]
Calnexin		Rabbit			500	200	Abcam Ab22595
γH2AX		Mouse		JBW301		250	EMD Millipore
Myc		Mouse	IgG1	9E10	5000	1000	Millipore
HA		Rat	IgG1	3F10	1000	500	Sigma
V5		Mouse	IgG2a		1000	1000	Invitrogen R960-25
GAPDH		Rabbit	Polyclonal				*Abcam ab9485*

Supplementary Table S2. List of secondary antibodies used

Target	Conjugation	Use	Dilution IF	Dilution WB	Source
anti-Mouse IgG1	Alexa Fluor 568	IF	500		Life Technologies
anti-Mouse IgG2a	Alexa Fluor 647	IF	500		Life Technologies

anti-Rat IgG1	Alexa Fluor 488	IF	500		Life Technologies
anti-Rabbit	Alexa Fluor 647	IF	500		Life Technologies
anti-goat	Alexa Fluor 488	IF	500		Life Technologies
Anti-mouse	HRP	WB		10,000	Dako
Anti-Rat	HRP	WB		10,000	Dako
Anti-Rabbit	HRP	WB		10,000	Dako

Supplementary Table S3. Primers used in this study

**Primer Name**	**Sequence**	**Purpose**
α-DG fw	AGGATGTCTGTGGGCCTCTCGCTGCT	Amplification cDNA dystroglycan
β-DG rev	AGGTGGGACATAGGGAGGAGGTGA	Amplification cDNA dystroglycan
V5 insert FW	GGTAAGCCTATCCCTAACCCTCTCCTCGGTCTCGATTCTAC-GGTGGGTGACCCGGCACTGGTGTTG	Overlap PCR: insertion V5-tag
V5 insert RV	CGTAGAATCGAGACCGAGGAGAGGGTTAGGGATAGGCTTACCAAACTT-GGCCTTGAACCTTGCAG	Overlap PCR: insertion V5-tag
HA insert FW	TACCCATACGATGTTCCAGATTACGCTAGGAGAGTGCCCTCAGAGGC	Overlap PCR: insertion HA-tag
HA insert RV	CAGTTTATCCCTGTGGTACCACCCTACCCATACGATGTTCCAGATTACGCT	Overlap PCR: insertion HA-tag
Myc insert FW	GAACAAAAACTCATCTCAGAAGAGGATCTGATGGGAGAGTACACGCCCCTG	Overlap PCR: insertion myc-tag

Myc insert RV	CAGATCCTCTTCTGAGATGAGTTTTTGTTCGGTGTCCTGGTTCAGAGGAGTG	Overlap PCR: insertion myc-tag
FL β-DG trunc.	TCCTGCAGGCGCGCCCGCCACCATGTCCATCGTGGTGGAATGGACCAACA	DG truncation: HAβmyc-DG
β-DG (cyto) trunc.	TGCGCAAGGCGCGCCCGCCACCATGCGCAAGAAGCGGAAGGGC	DG truncation: βmyc-DG (cyto)
DAG1 Cas9 FW seq	AAGGAGTTGCACGAAACTGTTGGTT	PCR Cas9 target region
DAG1 Cas9 RV seq	GAACACTGATTTAGGGATTTGTTGGA	PCR Cas9 target region

Supplementary Table S4. List of plasmids used / generated

**Plasmid name**	**Source**
pX458-gRNA-DG1	[59]
pX458-gRNA-DG2	[59]
pcDNA3.1-αV5-HAβmycDG	This study
pcDNA3.1-αV5-βDG	This study
pcDNA3.1-α-HAβDG	This study
pcDNA3.1-α-βmycDG	This study
pcDNA3.1-delta-α–HAβmycDG	This study
pcDNA3.1-βmycDG (cyto)	This study
pcDNA3.1-α-HAβmycDG	This study

SF1

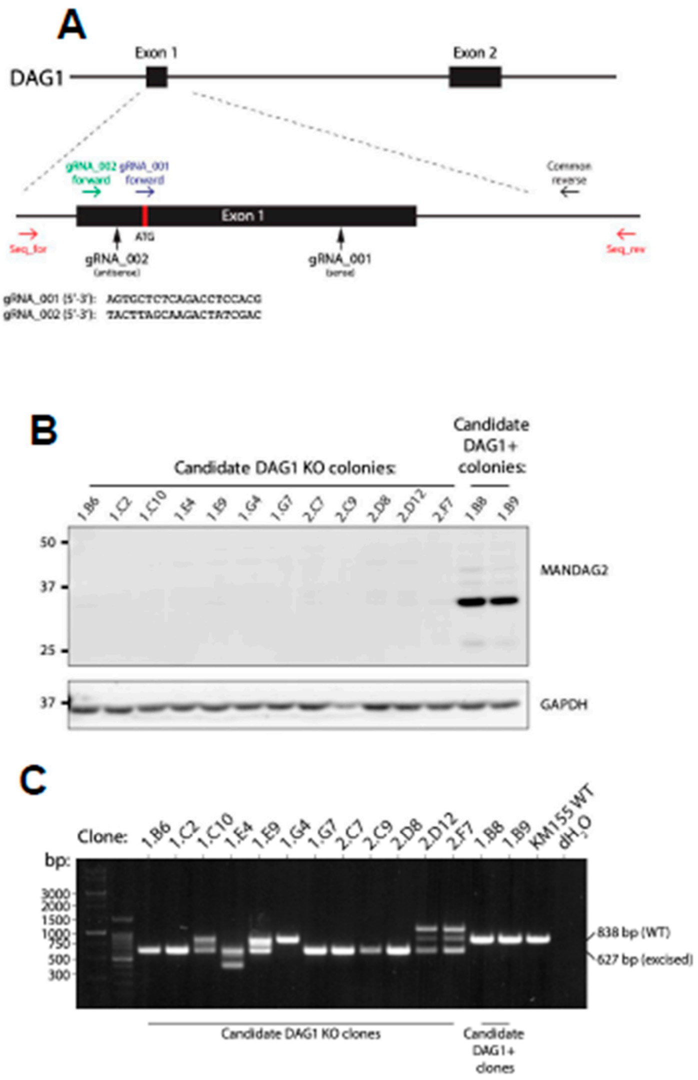

Supplementary Figure S1: Generating DAG1 KO KM155 myoblasts using CRISPR/Cas9.

A. DAG1 CRISPR KO strategy - schematic representation of DAG1 gene structure, indicating start codon (ATG) and positions of the targets for gRNAs. PCR primers (seq-for and seq-rev) and sequencing primers (gRNA_002 and gRNA_001 for) are also displayed.

B. Putative DAG1 KO clones were harvested from cell pellets and analysed by western blotting and probing for βDG using MANDAG2.

C. Genomic DNA was extracted and PCR amplified using seq-for and seq-rev primers in DAG1 KO clones and WT controls, and separated by agarose gel electrophoresis. Bands were gel extracted and cloned into TOPO-TA vectors for Sanger sequencing analysis of mutation induced and zygosity.

SF2

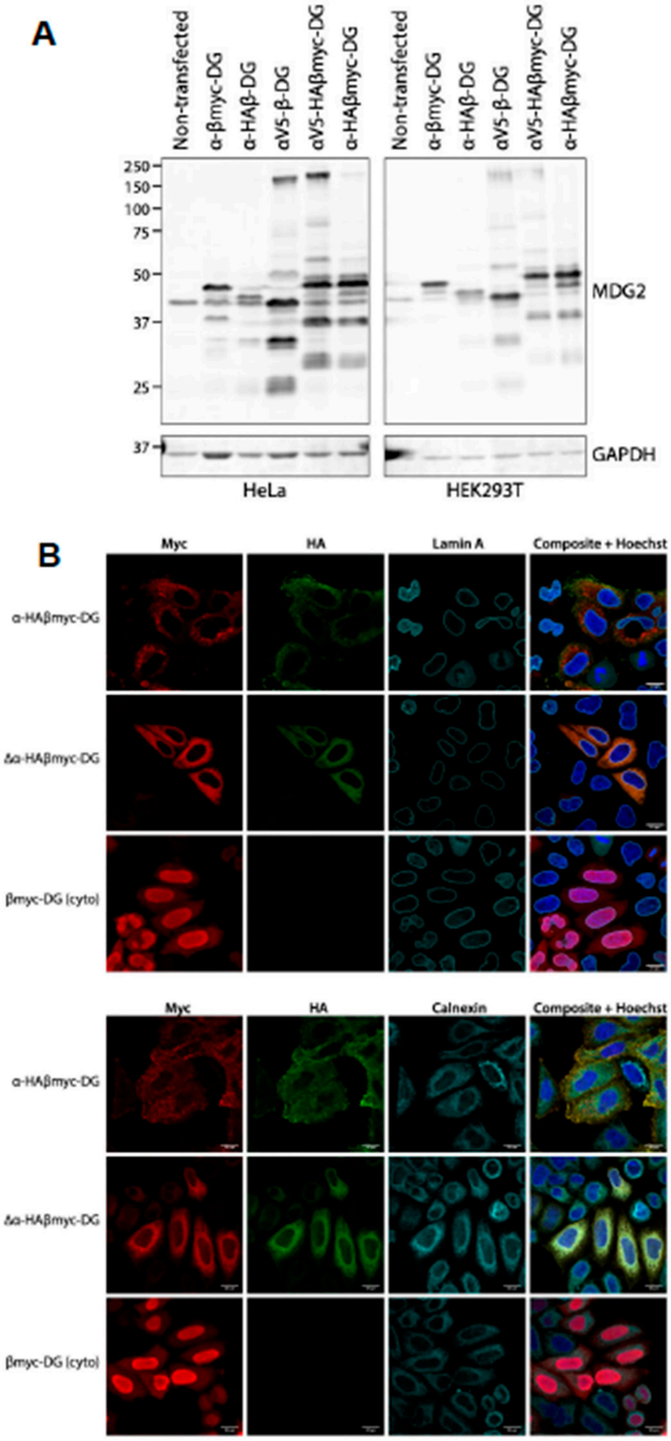

Supplementary Figure S2: αDG V5 tag placement impedes autoproteolysis

A. αDG V5 epitope tag placement impedes autoproteolysis. Epitope-tag placement in the carboxy-terminus of α-dystroglycan and throughout β-dystroglycan. HeLa and 293T were transfected with dystroglycan constructs containing indicated epitope tags and subject to SDS- PAGE. High molecular weight dystroglycan bands were detected using the MANDAG2 antibody.

B. Expression of dystroglycan fragments results in differential localisations. HeLa cells were transfected with either α-HAβmyc-DG, Delta-α-HAβmyc-DG or βmyc-DG (cyto), and stained with indicated antibodies to detect the epitope tags. Subcellular structures were also stained; the nuclear envelope was delineated with a stain for lamin A (upper), while the ER network highlighted with calnexin (lower). Scale bar = 20 µm

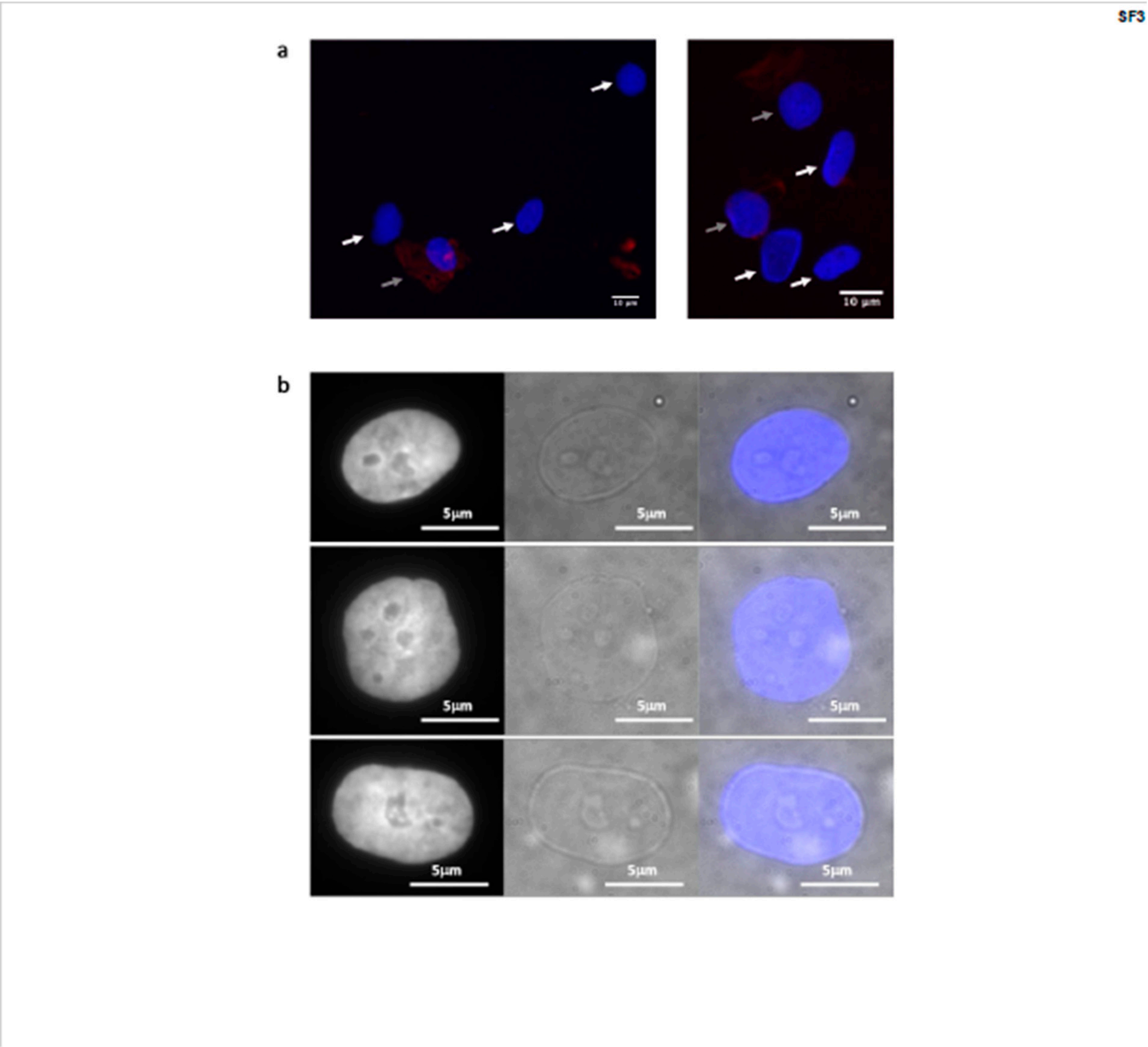

Supplementary Figure S3: Validation of nuclear isolation.

A. Representative nuclear isolation preparation stained with DAPI and phalloidin to identify the nucleus and any surrounding actin filaments indicative of cytoplasmic contamination. Cells lacking any phalloidin staining are indicated with white arrows while cells with some phalloidin staining remaining are indicated with grey arrows. Scale bar = 10 µm.

B. Representative brightfield microscopy images of isolated nuclei with no surrounding cytoplasmic contaminants. Scale bar = 5 µm.

SF4

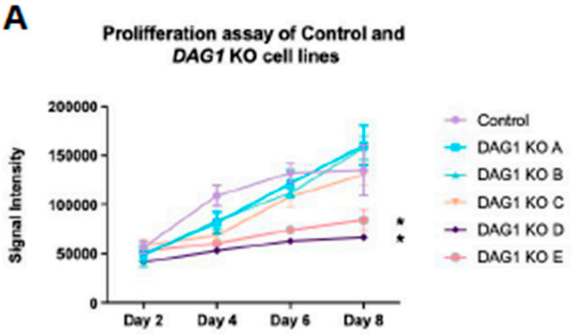

Time	KO A	KO B	KO C	KO D	KO E
**Day 2**	0.6574	0.7249	0.9858	0.1090	0.9169
**Day 4**	0.1091	0.1034	0.0315	0.0256	0.0379
**Day 6**	0.722	0.1311	0.1366	0.0158	0.0170
**Day 8**	0.6955	0.7061	0.9977	0.9999	0.3537

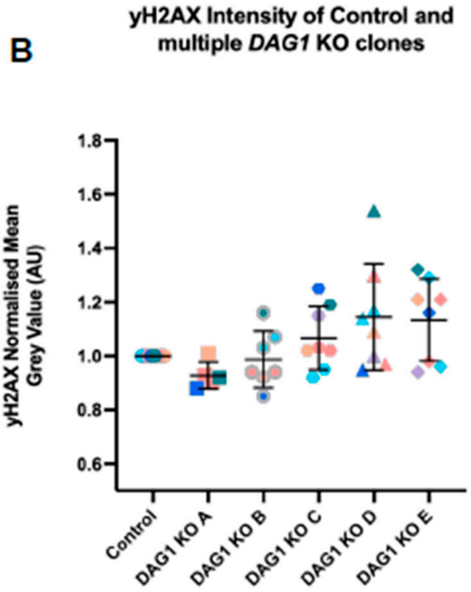

Cell Line	Average	SD	P value compared with control	Significance
Control	1.000	N/A	N/A	N/A
DAG1 KO A	0.9280	0.0497	0.3248	ns
DAG1 KO B	0.9871	0.1052	0.8455	ns
DAG1 KO C	1.054	0.1215	0.3019	ns
DAG1 KO D	1.166	0.2039	0.0276	*
DAG1 KO C	1.161	0.1414	0.0412	*

Supplementary Figure S4: DAG1 KO human myoblasts appear not to be senescent.

A. Cells were seeded and allowed to grow for 2, 4, 6 or 8 days when they were fixed. Cells were then stained with DRAQ5 to stain cell nuclei and the signal intensity of DRAQ5 was used as a measure of proliferation. 3 independent experiments were carried out. Points shows mean and standard deviation. Only DAG1 KO clones D-E grew significantly slower than the control cells while there was no statistically significant difference between control and DAG1 KO clones A-C. Statistical analysis used two-way ANOVA with Dunnett's multiple comparisons test, p values in the table.

B. Cells were plated 24 hours before fixing and staining with DAPI and an antibody against γH2AX to identify the levels of DNA damage. Cells were imaged using a Leica fluorescence microscope and the nuclear γH2AX signal intensity was determined for analysis. Each coloured point indicates the average for each independent experiment which was normalised to the control value. 5 independent experiments were carried out with 179 control, 179 KO A, 183 KO B, 188 KO C, 162 KO D and 150 KO E nuclei measured. Graph shows mean and standard deviation. A significant difference was determined between the samples as determined by one-way ANOVA test, p = <0.0179. p values for multiple comparisons using Dunnett's multiple comparisons test are in the table.

SF5

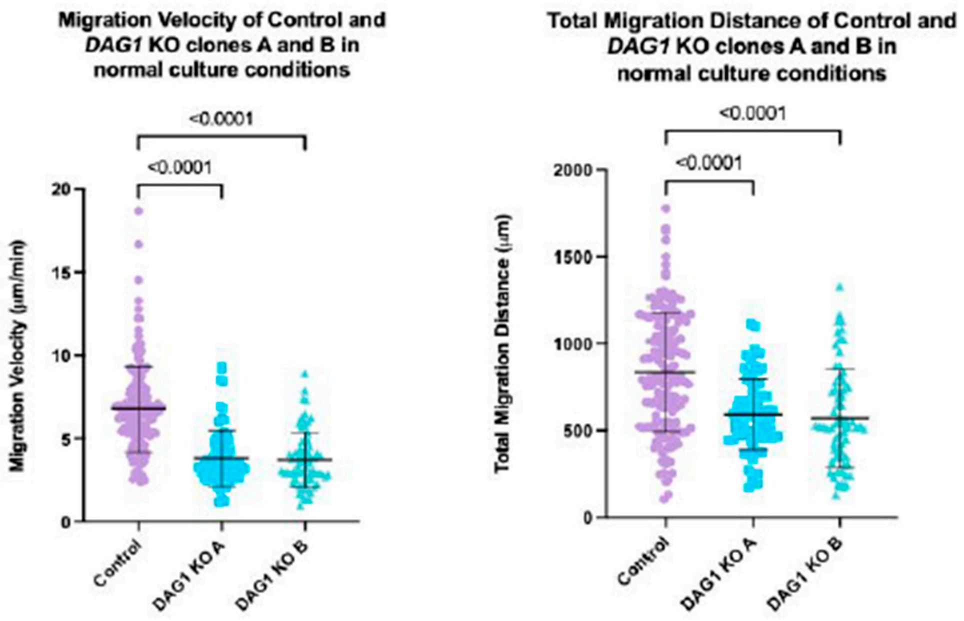

Supplementary Figure S5: Migration velocity and migration distance of DAG1 KO myoblasts

Migration velocity and migration distance of DAG1 KO myoblasts under normal culture conditions. (left) Cell velocity; (right) Total migration distance in 16 hours. Each point indicates an individual cell. 2 independent experiments were carried out with 160 control, 150 KO A and 143 KO B cells measured. Graph shows mean and standard deviation. The one-way ANOVA test with Dunnett's multiple comparison test.

References

57. Ilsley, J.L.; Sudol, M.; Winder, S.J. The interaction of dystrophin with β-dystroglycan is regulated by tyrosine phosphorylation. Cell. Signal. 2001, 13, 625–632.
58. Pereboev, A.V.; Ahmed, N.; Man, N.T.; Morris, G.E. Epitopes in the interacting regions of β-dystroglycan (PPxY motif) and dystrophin (WW domain). Biochim. Biophys. Acta—Gen. Subj. 2001, 1527, 54–60. https://doi.org/10.1016/s0304-4165(01)00147-7.
59. Ran, F.A.; Hsu, P.D.; Wright, J.; Agarwala, V.; Scott, D.A.; Zhang, F. Genome engineering using the CRISPR-Cas9 system. Nat. Protoc. 2013, 8, 2281–2308. https://doi.org/10.1038/nprot.2013.143.
60. Rivier, F.; Robert, A.; Hugon, G.; Bonet-Kerrache, A.; Nigro, V.; Fehrentz, J.-A.; Martinez, J.; Mornet, D. Dystrophin and utrophin complexed with different associated proteins in cardiac Purkinje fibres. Histochem. J. 1999, 31, 425–432. https://doi.org/10.1023/a:1003805905456.
